# Brownfield Topsoil Vertical Heterogeneity: Implications for Germination and Soil Microbial Functioning

**DOI:** 10.1021/acsomega.4c05265

**Published:** 2024-09-25

**Authors:** Eshariah N. Dyson, Diane F. Hagmann, Cesar Idrovo, Jennifer Adams Krumins, Nina M. Goodey

**Affiliations:** † Department of Chemistry and Biochemistry, Montclair State University, Montclair, New Jersey 07043, United States; ‡ Department of Earth and Environmental Science, Montclair State University, Montclair, New Jersey 07043, United States; § Department of Biology, Montclair State University, Montclair, New Jersey 07043, United States

## Abstract

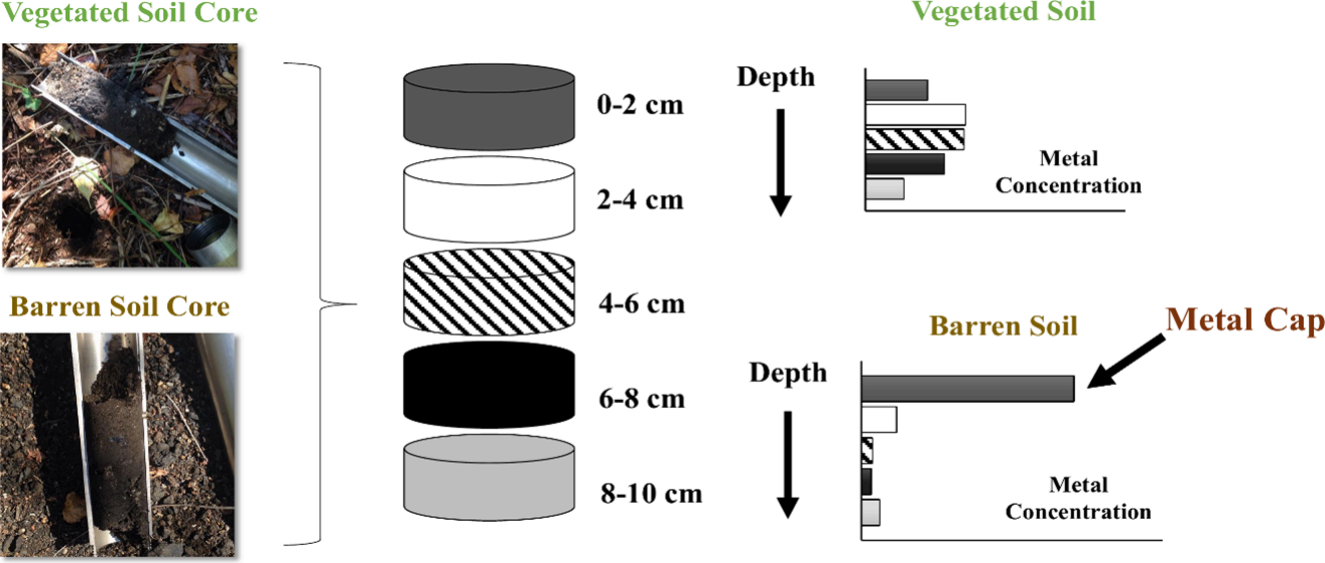

Soil vertical heterogeneity refers to the variation in soil properties and composition with depth. In uncontaminated soils, properties including the organic matter content and nutrient concentrations typically change gradually with depth due to natural processes such as weathering, leaching, and organic matter decomposition. In contaminated soils, heavy metals and organic contaminants can migrate vertically through leaching or root uptake and translocation by plants and macrobiota, if present, leading to vertical heterogeneity in contaminant concentrations at different depths. Contaminants can alter soil properties, and we investigated the implications of soil vertical heterogeneity for germination and microbial functioning. We collected soil from an urban brownfield and created two conditions: structured soil samples collected with the soil core intact and mixed (unstructured) samples. When planted, the germination rate was significantly lower in the structured conditions (3.1 ± 1.7%) compared to mixed soils (17 ± 4.6%), suggesting that the vertical heterogeneity of contaminated soil influenced plant germination. To map the vertical distribution of contaminants and nutrient cycling rates in the structured soil samples, we collected 10 cm-deep soil cores from the barren site and a neighboring vegetated reference site and measured heavy metal concentrations, soil enzyme activities, and organic matter content in five 2 cm vertical layers. In the barren soil cores, metals were found concentrated in the top 2 cm layer, while in the vegetated soil cores, metals were uniformly distributed. No significant differences were observed for the organic matter content or moisture along depth. Published studies on vertical distribution of enzyme activities and metal concentrations have treated the top 10–20 cm as a single layer and thus would have not revealed the thin (<2 cm thick) metal cap on the surface of the barren soil core. Despite the metal cap, enzyme activities in the top layer were similar to those in the lower layers of the barren soil core, suggesting that high metal concentrations do not limit soil enzyme activity under all circumstances. Investigating vertical heterogeneity in postindustrial soils can inform efforts to convert industrial barrens to vegetated environments.

## 1 Introduction

Soil is an important nonrenewable resource that plays a major role in food production, water filtration, and carbon and nitrogen cycling. Soil heterogeneity refers to the nonuniform vertical and horizontal distribution of soil components.^1^ Typical undisturbed soils exhibit vertical soil horizons with organic matter at the surface followed by a layer containing clays, oxides, and carbonates and a bottom layer containing salts.^2^ Human augmentation of soils can drastically affect soil properties and morphology.^3^ Many urban soils are influenced by human activity and differ from naturally developed soils, typically containing a layer of fill that originates from building materials, clays, and sand and could be contaminated.^2^ This fill layer may sit on top of existing soil and result in “buried” soil horizons and influence the migration of water and contaminants through the soil.^4,5^ Brownfield soils also contain contaminants that can negatively impact soil function and nutrient cycling.^6^ For example, heavy metals can cause shifts in soil microbial communities and alter microbial activity.^7^ Contaminants can also limit plant germination and productivity.^6,8^ There are numerous examples of soils throughout the world that have become barren as a result of heavy metal contamination. For example, areas in the Arctic Kola Peninsula near the Severonikel smelter in Monchegorsk, Russia were heavily impacted by industrial activities that have resulted in accumulation of bioavailable heavy metals and soil degradation. The accumulation of metals such as Ni, Cu, and Co has led to nutrient imbalances and the death of plants. Both birch (*Betula pubescens*) and willow (*Salix caprea*) near the Severonikel smelter have been shown to exhibit significant nutrient deficiencies and accumulate toxic metals.^9^ Kozlov and Zvereva reported on a site near Monchegorsk where heavy metal inputs from the Severonikel smelter resulted in nutrient depletion, low biological activity, and impaired plant growth. The extreme Arctic climate and continuous pollution further exacerbate soil degradation, preventing vegetation recovery at this site.^10^

Anthropogenic activity has become a major factor in soil formation, and the degradation of soil by anthropogenic interference is a global issue that has implications for human health, land usage, and the earth’s ecosystems.^11,12^ Contamination can render a soil an industrial barren, which is a contaminated, extreme environment that has little to no plant growth due to human activity.^13^ Industrial barrens are typically contaminated with inorganic and organic compounds and often remain abandoned for several decades. These areas present a unique opportunity to study the impacts of contamination on soil properties and plant germination and growth. Metal contamination found in industrial barrens has both physiological and biochemical effects on seed germination.^14^ Heavy metals can cause starch immobilization, nutrient limitations, and reduced proteolytic enzyme activities.^15^ In many contaminated sites, a combination of different metal contaminants are found in the soil, and multimetal contamination has been found to cause a reduction in seed germination.^16^ Studies on the effects of metals on enzyme activities have predominantly focused on Cu, Cd, Pb, Zn, Ni, and As.^14,15,17^ Studies on the effect of metals on germination have predominantly focused on Cu, Zn, Pb, Cd, and As.^18,19^ In this study, we focused on the metals Pb, Zn, Ni, As, Co, Ba, Cu, and V. Phytoremediation efforts present a cost-effective solution to revitalize barren soils but are dependent on the soil’s ability to support plant life.^20^ The literature on phytoremediation reports that high heavy metal concentrations are linked with poor fertility and a lack of organic matter can make revegetation difficult.^18^ When soils lose their ability to support plants, phytoremediation is no longer a possible solution.

The relationship between metal concentration and soil depth has been investigated.^21,22^ The studies typically focus on depths spanning from the soil surface to about 150 cm and provide valuable information about metal distributions in deeper soils.^23,24^ However, a significant fraction of microbial activity, including decomposition and mineralization of nutrients, occurs in the top ∼10 cm of the soil due to factors including accumulation of organic matter, moisture, and soil microfood-web activity.^25,26^ Prior research on soil parameters through depth has divided soil into large layers that are greater than 10 cm. Here, we evaluated, at a finer level of resolution, the properties of thinner (two cm) vertical layers in the top 10 cm of the soil, the soil depth where microbial activity is most relevant for plant productivity.^27^ We chose the 10 cm depth because it is predominantly in this region where plant roots first come in contact with soil and where they interact with soil microbes.

**Figure 1:**
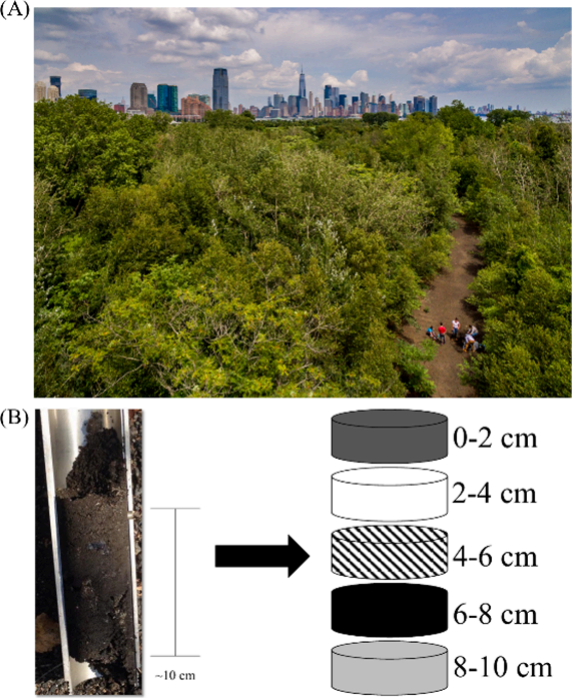
(A) Aerial view of the closed-off LSP study area with the vegetated site (25F) and barren site (25R) shown. (B) Photo of a soil core shown along with a cartoon that illustrates how the core was divided into layers. Photograph courtesy of Mike Peters/Montclair State University. Copyright 2024.

Liberty State Park (LSP) is an unremediated brownfield in Jersey City in northern New Jersey, USA. Once used as an industrial railyard, it was abandoned in the 1960s. The area remained undisturbed for about 50 years before most of the land was converted into a park for recreational use. A large area of the park, contaminated with both heavy metals and organic contaminants, remained unremediated and fenced off from the public. Our two study sites within this area have similar types of contaminants and, as adjacent sites, were exposed to similar climate conditions.^28^ Interestingly, one site within the park remained an industrial barren (25R), while the adjacent site took a trajectory that resulted in vegetation and a lush forest (25F) (Figure 1A). In this manuscript, we used the previously established site names/numbers (for example, 25R and 25F) from published work.^29^ A forest surrounds barren site 25R, which has been undisturbed for over 60 years, but surprisingly the site remains barren today. These sites present an opportunity to study how soil vertical heterogeneity varies between vegetated and barren brownfield soils.

In a controlled laboratory study, we compared plant germination and primary production in structured and mixed barren, contaminated soils. We also measured the relationship between soil depth, metal concentrations, and enzyme activities within the top 10 cm of the soil collected from vegetated and barren LSP sites located adjacent to each other (Figure 1B). The study design allowed us to compare vertical distributions of soil properties including metal concentrations, extracellular soil enzyme activities, organic matter, and moisture (top 10 cm) in contaminated vegetated and barren soils. One of the objectives of this study was to determine the impact of vertical heterogeneity in a barren, contaminated soil on plant germination rates by comparing structured and mixed soils. Additionally, the study aimed to assess the differences in the vertical distributions of soil properties between vegetated and barren contaminated soils.

## 2 Methods

### 2.1 Study Site and Soil Preparation

LSP began as a railyard, through which trains transported goods and people between the mid-Atlantic and New York City, primarily in the 19th century. Post abandonment, most of the site was remediated into the urban park present today, but a 100 ha site was closed off from the public, allowing for naturally occurring ecological succession.^30,31^ Soil at LSP has been given its own designation by the USDA, the Lady Liberty Series.^8^ Our study sites, 25F and 25R, are located inside this closed-off 100 ha area, share a similar industrial history, and, to our knowledge, have not been plowed or impacted by any anthropogenic activity since the 1970s (Figure 1A).

Soils at these sites have heavy metal concentrations that exceed clean-up criteria and background metal concentrations at a comparable, forested New Jersey site.^7^ A barren site (25R) at LSP has high heavy metal concentrations and poor enzymatic function. Yet, these high concentrations have not explained why this site is barren because an adjacent reference site (25F) is vegetated, has high enzymatic function, and similar organic and inorganic contaminants.^28,32^Table S1 shows a comparison between individual metal concentrations in 25R and 25F soils. Both sites have the same metals present, the concentrations of some of the metals are higher in 25R, while the concentrations of others are higher in 25F. Site 25R is very slightly elevated compared to 25F, and water may flow from 25R into 25F due to this small elevation difference.^8^ Bulk 25R soil has a pH of 5.04, an organic matter content of 23 ± 0.2%, an aggregate size *d* (0.5) of 443 ± 30 μm, and a water holding capacity of 29 ± 0.8% w/w. Bulk soil samples from a comparative vegetated site (site 146) within LSP have a pH of 5.20, an organic matter content of 43 ± 0.5%, an aggregate soil size *d* (0.5) of 105 ± 4 μm, and a water holding capacity of 133 ± 0.9% w/w.^32^

### 2.2 Inorganic Elements

To study the vertical distribution of metal contaminants at the site, soil core samples were collected from site 25R and a vegetated reference site 25F in LSP. Three replicate 10 cm-deep soil cores, 10 m apart from one another, were removed from each of the two study sites, using a 5 cm-wide stainless-steel slam bar soil core. Each core was divided through depth into five 2 cm layers (0–2, 2–4, 4–6, 6–8, and 8–10 cm, Figure 1B). Each layer was separated then passed through a 2 mm sieve, bagged, and stored in a refrigerator at 4 °C.

To determine metal concentrations of each layer, we followed the protocol outlined by Hagmann et al.^8,28^ Soil samples (0.5 g) were dried (∼100 °C) for 24 h, ground to a fine powder, and then placed in a 50 mL digestion tube. The soils were digested following EPA method 3050B.^28^ Briefly, 2.5 mL of a 1:1 nitric acid solution was added to the digestion tube, and the sample was refluxed for 15 min at 95 °C. After cooling, concentrated nitric acid (5 mL) was added, and the solution was refluxed for an additional 30 min at 95 °C. While refluxing, the solution was monitored for the formation of brown fumes. Once the fumes stopped forming, the solution was maintained at the same temperature (95 °C) until the volume reduced to 2.5 mL. Cooled solutions were then filtered (1 μm virgin polypropylene DigiFilter, SCP Science, Quebec, Canada) to remove any remaining soil solids, diluted, and analyzed for metal concentrations on an inductively coupled plasma mass spectrometer (ICP-MS, Thermo Fisher Scientific, Bremen, Germany). The metals measured for each soil site were Pb, Zn, Ni, As, Co, Ba, Cu, and V. A seven-point standard curve was determined for each metal using dilutions of commercial stock solutions in 5% HNO_3_. A blank (5% HNO_3_) was used as a negative control in each experiment. The detection limit for the instrument was <1 ppb.

### 2.3 Plant Germination Rates in Mixed and Structured 25R Soils

To determine if the vertical soil heterogeneity of contaminants in barren site 25R affects the rate of germination, soil was collected in a grid pattern alternating between structured (collected in PVC columns) and mixed (scooped into a ziploc bag) soil with a 1 m spacing between each sample. For this study design, because agitation of the soil during transport was possible, we chose a large number of replicates (*n* = 16 for structured and *n* = 13 for mixed). We originally planned to have 16 replicates of both the structured and mixed samples but due to the soil available for this experiment, we were only able to achieve *n* = 13 for the mixed samples. Structured samples were collected from the site by hammering 2 in.-wide polyvinyl chloride (PVC) columns about 15 cm into the ground then removing it intact, thus maintaining integrity of the layered structure. These became the structured soil samples. As a control, mixed soil samples were assembled by filling parallel PVC columns with stirred soil with no layered structure. The soil was poured into the PVC columns and allowed to settle on its own. It is important to be aware that this repacking could have affected the pore size and therefore the hydraulic conductivity of the soil. The key assumption was that mixing the soil resulted in homogenization of soil properties. Each column received six premium winter rye grass seeds (*Lolium perenne*), a horticulturally available plant that is naturally available at the site, and watered (5 mL, sterile tap water) three times a week for 6 weeks. Columns were kept in an incubator with controlled environmental conditions on a 10.5/13.5 h day/night cycle. The relative humidity was 65%, and the temperature was 24 °C for the day cycle and 16 °C for the night cycle.^32^ Photos were taken every week to monitor plant growth in the columns. At the end of 6 weeks, plant shoots were harvested, and germination rates (shoots/seeds sown), shoot heights (cm), dried shoot masses (g), and dried root masses (g) were determined.

To test the germination rates in soils collected from the top 0–2 cm and the bottom 8–10 cm layers, 2–3 g of soil was added to a small weighing boat. The same 25R soil that was collected for other measurements including metal concentrations, enzyme activity, and moisture from the 0–2 cm and 8–10 cm layers was used for this experiment. The soil was collected from three replicate 10 cm-deep soil cores located 10 m apart from one another in a linear transect. Six winter rye grass seeds were added to the soil in each weighing boat, and they were placed in a well-lit area for 21 days. Three replicate experimental units were set up for each soil layer. The soil was watered every other day to maintain soil moisture. At the end of 21 days, the plants were counted, and the germination rates for each individual experimental unit (weighing boat) were calculated.

### 2.4 Enzyme Assays

We measured phosphatase, cellobiohydrolase, l-leucine amino peptidase, and peroxidase activities for layers from the side-by-side vegetated and barren sites 25F and 25R. The first three extracellular enzyme activities are involved in P, C, and N mineralization, respectively.^8,33−35^ Peroxidase, on the other hand, has been shown to play a role in relieving oxidative stress in metal-contaminated soils.^36^

#### 2.4.1 Phosphatase

To measure phosphatase activity, we followed the protocol outlined by Marx and co-workers.^37^ Three wells contained 350 μM of the substrate 4-methylumbelliferyl-phosphate. Soil (0.1 g) was mixed into MES buffer solution (100 mL, 0.1 M, pH = 6.1) and sonicated (Fisherbrand model 505 sonic dismembrator, Fisher Scientific, Parsippany, New Jersey) at a 30% amplitude for 3 min. Standard curves had 0, 500, 1300, 2000, 3500 pmol of the product (4-methylumbelliferone) in four different wells. Phosphatase assays were run at 30.0 °C with an excitation wavelength of 320 nm and an emission wavelength of 450 nm. The reaction was run in a black bottom microtiter assay plate for 3 h on a fluorometer (BioTek Synergy H1 hybrid multimode reader, BioTek, Winooski, Vermont), with readings after every 7.5 min.

#### 2.4.2 Cellobiohydrolase

To measure cellobiohydrolase activity, we followed the protocol outlined by Hagmann et al.^8^ Three wells in a black bottom microtiter assay plate contained 650 μM of the substrate (4-methylumbelliferyl-d-cellobioside) in a total volume of 200 μL. Soil (0.5 g) was mixed into an MES buffer (100 mL) and sonicated at a 30% amplitude for 3 min. Standard curves had 0, 50, 100, 250, and 500 pmol of the product (4-methylumbelliferyl) in four different wells with a total volume of 200 μL. Cellobiohydrolase assays were run at 30.0 °C with an excitation wavelength of 320 nm and an emission wavelength of 450 nm. The reaction was run for 6 h on a fluorometer, with readings taken every 15 min.

#### 2.4.3 l-Leucine Amino Peptidase

To measure l-leucine amino peptidase activity, we followed the protocol outlined by Hagmann et al.^8^l-Leucine-7-amino-methylcoumarin was used as a substrate at a concentration of 400 μM in each well with a total volume of 200 μL. Soil (0.5 g) was mixed with an MES buffer (100 mL) and sonicated at a 30% amplitude for 3 min. Standard curves were created with 0, 800, 1200, 1800, and 2400 pmol of the product (4-amino-7-methylcoumarin) in four different wells with a final volume of 200 μL. l-Leucine amino peptidase assays were run at 30.0 °C with an excitation wavelength of 350 nm and an emission wavelength of 440 nm. The reaction was conducted in a black bottom microtiter assay plate for 3 h, with readings taken every 7.5 min.

#### 2.4.4 Peroxidase

To measure peroxidase activity, we followed published protocols by Bach et al. and Sinsabaugh.^36,38^ Briefly, an acetate buffer (50 mM, pH 6.1) was prepared, using sodium acetate and glacial acetic acid to adjust the pH. A soil slurry was prepared by combining soil (1 g) with the acetate buffer (125 mL). The soil slurries were sonicated at a 25% amplitude and constant pulse for 3 min. While the slurry was mixing continuously, aliquots of slurry (200 μL) were added to the wells in a clear 96-well microplate.

The substrate l-3,4-dihydroxyphenylalanine (L-DOPA) was dissolved in hot, deionized (DI) water at about 90 °C to make a 25 mM solution. In addition to the soil slurry, sample wells also contained 0.3% H_2_O_2_ (10 μL) and 25 mM L-DOPA (50 μL). To correct for the substrate background, blank wells containing 0.3% H_2_O_2_ (10 μL), 50 mM acetate buffer (200 μL), and 25 mM L-DOPA (50 μL) were used. To correct for the sample background of each soil sample, negative control wells containing soil slurry (200 μL), 0.3% H_2_O_2_ (10 μL), and 50 mM acetate buffer (50 μL) were used. Absorbance values measured at 460 nm were obtained using a microplate reader running for 3 h, with readings after every 7.5 min. To determine the peroxidase activity, the average absorbance values of the blank and the negative control were subtracted from the average absorbance value of each soil sample and divided by the extinction coefficient of 7.9 mM ^–1^cm^–1^.

A Michaelis–Menten plot was constructed to determine the best concentration of the L-DOPA substrate to use for the peroxidase assay. A microplate assay was performed following the peroxidase protocol stated above with only vegetated 25F soil and varying substrate concentrations from 0 to 35 mM. The formation of the product was monitored for every 7.5 min over 3 h, and the peroxidase activity was calculated for each substrate concentration.

### 2.5 Percent Moisture and Organic Matter Content

The percent moisture content of each soil layer was determined by weighing out soil (1 g) followed by heating at 100 °C for 24 h. After cooling, the soil was weighed again. The percent moisture was determined gravimetrically in triplicate.^32^ The organic matter content of each layer of the soil was determined by loss on ignition. Homogenized dry soils (100 °C) from each layer were placed in a muffle furnace and heated to 550 °C for 4 h. The organic matter content was determined gravimetrically.^30^

### 2.6 Data Analysis

Data for soils collected from vegetated site 25F were analyzed separately from data for barren site 25R. An analysis of variance (ANOVA) was conducted between layers to compare enzymatic activity, organic matter, moisture, and concentrations of inorganic elements with a significance cutoff value of *p* < 0.05. A log transformation was applied to the response variables to reduce heteroscedasticity. Where significant effects were found, a post hoc test (Tukey’s HSD) was conducted. Spearman correlations were used to analyze possible relationships between enzyme activities, organic matter, and concentrations of inorganic elements using the Hmisc package with a significance cutoff value of *p* < 0.1.^39^ A paired *t* test was used to compare the means of the germination rates, shoot heights, shoot masses, and root masses of plants potted in mixed and structured barren 25R soil. A paired *t* test was used to compare the germination rates in the top 0–2 cm and bottom 8–10 layers of 25R soil. All statistics were performed using R (version 3.6.2).^40^

## 3 Results

### 3.1 Inorganic Elements Accumulated on the Barren 25R Soil Surface

**Figure 2:**
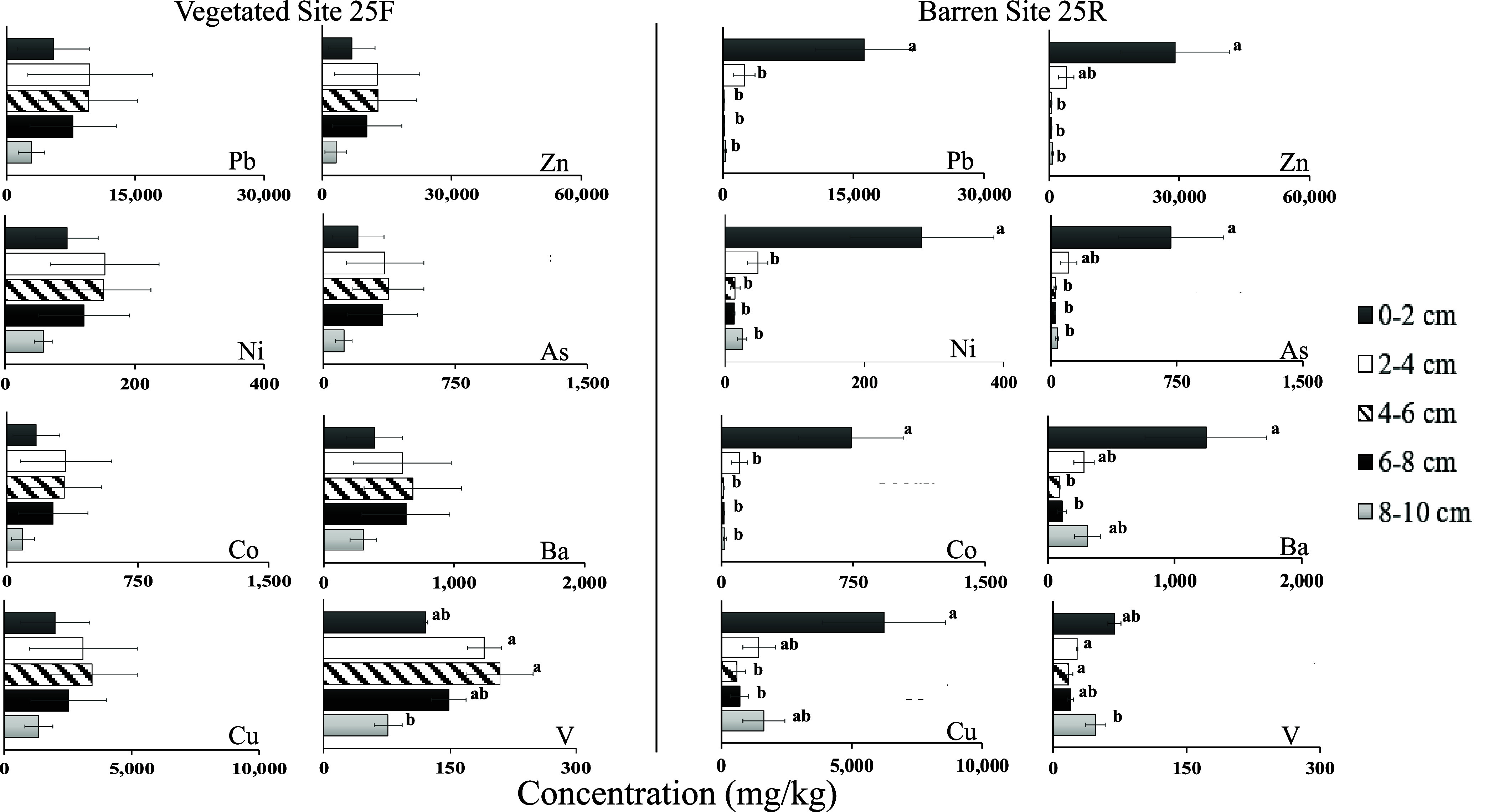
Pb, Zn, Ni, As, Co, Ba, Cu, and V concentrations (mg/kg) in five soil layers from vegetated site 25F (left) and barren site 25R (right). The bars representing means and standard errors are shown (*n* = 3). Note that the *x*-axis value ranges differ among metals. Means denoted by different letters indicate significant differences among the layers (Tukey’s test, *p* < 0.05).

At the vegetated reference site 25F, metal concentrations did not vary with soil depth except for V (*F*_4,10_ = 6.1, *p* = 0.0093) (Figure 2). In contrast, at barren site 25R, the concentrations of seven of the measured metals (Pb, Zn, Ni, As, Co, Ba, and Cu) were significantly higher in the top layer (0–2 cm) compared to deeper layers, indicating that metals accumulated on the soil surface (Figure 2). For example, the Pb concentration was significantly higher in the top 0–2 cm layer (16,200 ± 5575 mg/kg) compared to the bottom layer (301 ± 114 mg/kg) (*F*_4,10_ = 15.7, *p* = 0.0003). Vanadium was the only metal for which we did not observe a statistically significant difference between the top and bottom layers in 25R. Vanadium has several oxidation states, is heavily influenced by the soil redox potential, and tends to form complexes with organic matter.^41^ These factors may help explain the different vertical distribution of vanadium compared to the other metals. Similar metal concentration trends along depth were observed for each of the replicate cores collected along a transect 10 m apart within each site, suggesting that the trends with depth were similar across each site.

### 3.2 Plant Growth in Mixed and Structured 25R Soils

In the mixed columns of 25R soil, 12 winter rye grass seeds germinated out of the 78 total seeds planted across the 13 mixed soil columns. Of the 96 total seeds planted across the 16 structured soil columns, only three plants germinated. It is possible that the structured columns experienced some agitation during transport by foot through challenging terrain at LSP even though we did our best to minimize this. This agitation may have been responsible for the three plants that were able to establish across the 16 structured columns although the plants may have alternatively germinated simply due to natural differences in vertical heterogeneity.

**Figure 3:**
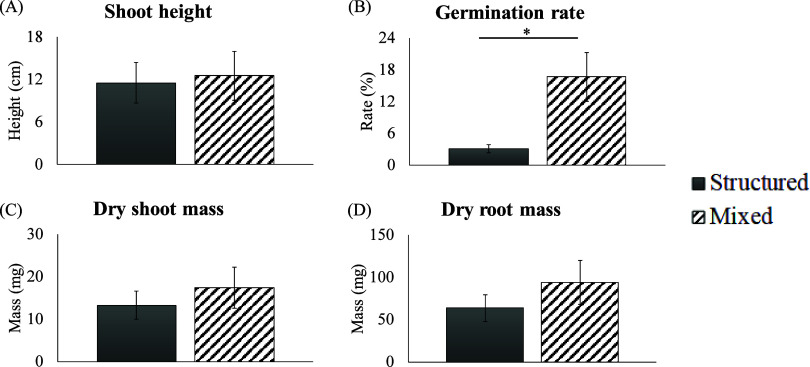
Shoot height (A), germination rate (B), dry shoot mass (C), and dry root mass (D) for structured and mixed PVC columns of 25R soil planted with winter rye grass seeds. The germination rate for each column was calculated as the number of shoots divided by the number of seeds sown. Columns with no shoots had a germination rate of zero, and these zeroes were included in the calculation of the means. The germination rate was significantly lower in structured compared to mixed PVC columns (*t* = 2.753, df = 15.17, and *p* = 0.0146). The star (*) indicates a significant difference (*p* < 0.05).

The germination rate for each column was calculated as the number of shoots divided by the number of seeds sown into that column (each column received six seeds). Columns with no shoots had a germination rate of zero, and these zeroes were included in the calculation of the means. The germination rate in the mixed columns (17 ± 4.6%) was significantly higher than the germination rate in the structured soil columns (3.1 ± 1.7%) (*t* = 2.753, df = 15.17, and *p* = 0.0146) (Figure 3B). Germination rates of winter rye grass in both structured (3.1 ± 1.7%) and mixed (17 ± 4.6%) columns of contaminated 25R soil were lower than typical rates in uncontaminated soils, which are approximately 90%.^42^ This is not surprising because it has been shown that metal contamination, particularly a mixture of different metals in soils, can lead to decreased germination rates for winter rye grass.^43^ The shoot height (Figure 3A), dry shoot mass (Figure 3C), and dry root mass (Figure 3D) were also higher in the mixed columns, but only the difference in the germination rate was found to be statistically significant between the two conditions.

To compare the germination rates in soils collected from the top 0–2 cm and the bottom 8–10 cm layers, winter rye grass seeds were planted into these soils in weighing boats in laboratory conditions. Of the 18 total seeds planted in soil from the top 0–2 cm layer, 7 seeds germinated. Of the 18 seeds planted in soil from the bottom 8–10 cm layer, 12 seeds germinated. The germination rate in the 0–2 cm layer (39 ± 6%) was lower than in the 8–10 cm layer (67 ± 10%) (*t* = 2.5, df = 3.2, and *p* value = 0.08) (Figure S1). These results indicate that the top 0–2 cm layer of 25R soil is not conducive to winter rye grass germination. The results are in agreement with the germination rate data for the mixed and structured PVC columns, which showed that the undisturbed surface of the 25R soil is not conducive to winter rye grass germination (Figure 3B). We next set out to determine how metal concentrations, extracellular enzyme activities, organic matter content, and moisture varied among the vertical soil layers in the structured soil columns.

### 3.3 Phosphatase, Cellobiohydrolase, and l-Leucine Amino Peptidase Activities

**Figure 4:**
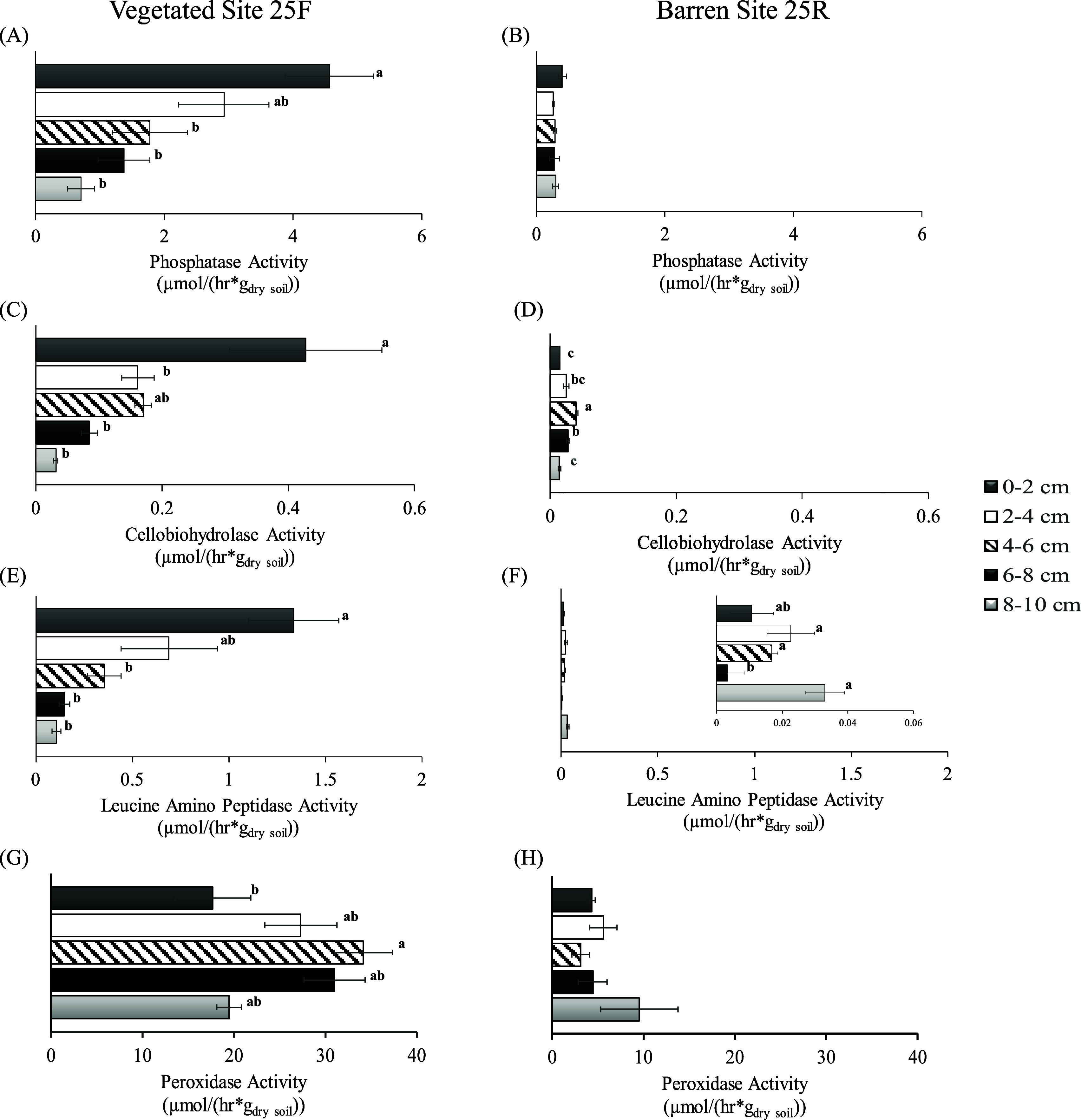
Extracellular soil phosphatase (A,B), cellobiohydrolase (C,D), l-leucine amino peptidase activity (E,F), and peroxidase activity (G,H) are shown for five vertical 2 cm soil layers from vegetated (25F, left) and barren (25R, right) sites. All enzymatic activities are reported as the means in μmol/(h g_dry soil_), *n* = 3, and standard errors are shown. Means denoted by different letters indicate significant differences between layers (Tukey’s test, *p* < 0.05).

Phosphatase, cellobiohydrolase, and l-leucine amino peptidase activities were lower in barren site 25R compared to the vegetated reference site 25F (Figure 4A–F). The enzyme activities did not vary systematically with depth in 25R soil. On the other hand, phosphatase, cellobiohydrolase, and l-leucine amino peptidase activities decreased with depth in 25F cores. The top 0–2 cm layer of the vegetated site 25F had the highest extracellular phosphatase (4.6 ± 0.7 μmol/(h g_dry soil_)), cellobiohydrolase (0.4 ± 0.1 μmol/(h g_dry soil_)), and l-leucine amino peptidase (1.3 ± 0.2 μmol/(h g_dry soil_)) activities (Figure 4A, C, and E). Spearman correlation analysis (Figure S2A) showed that the nutrient cycling enzyme activities (phosphatase, cellobiohydrolase, and l-leucine amino peptidase) in 25F soil were positively correlated with each other but were not significantly correlated with any of the metal concentrations in the soil. Conversely, in barren 25R, cellobiohydrolase activity showed a significant negative correlation with Zn (*r* = −0.54, *p* = 0.036), Pb (*r* = −0.45, *p* = 0.089), Ni (*r* = −0.45, *p* = 0.089), V (*r* = −0.65, *p* = 0.009), and Ba (*r* = −0.64, *p* = 0.0102). Moreover, the phosphatase activity in 25R soil had a significant positive correlation with Cu (*r* = 0.49, *p* = 0.062), V (*r* = 0.54, *p* = 0.038), and Ba (*r* = 0.54, *p* = 0.036) (Figure S2B). The data show that the relationships between enzyme activities and metal concentrations depend on the soil abiotic context and the specific enzyme activity that is measured.

### 3.4 Peroxidase Activities of Vegetated Soil 25F and Barren Soil 25R

To measure peroxidase activity in the top 10 cm of LSP soils, we first determined the substrate concentration required for the assay using Michaelis–Menten kinetics. Using the calculated peroxidase activity and the corresponding concentrations of L-DOPA, we constructed a Michaelis–Menten curve (Figure S3). The data were fitted to the nonlinear Michaelis–Menten equation, yielding a *V*_max_ of 22.5 ± 1.4 s^–1^ and a *K*_M_ of 0.29 ± 0.06 mM for L-DOPA. These data indicate that a 25 mM concentration of the L-DOPA substrate was more than sufficient to reach maximum velocity for peroxidase activity assays. We previously determined and reported the substrate concentrations required to reach maximum velocities for the other enzyme activities (phosphatase, cellobiohydrolase, and l-leucine amino peptidase) using soils from LSP.^8^

Layers from vegetated 25F soil had significantly different peroxidase activities (*F*_4,10_ = 3.8, *p* = 0.0395). Starting from the top layer, peroxidase activity increased with depth to a maximum value (34 ± 3.2 μmol/(h g_dry_)) in the 4–6 cm layer followed by a decrease for deeper layers (Figure 4G). The metal concentrations and peroxidase activities followed a similar trend among the vegetated 25F layers (Figures 2 and 4G). Spearman correlation analyses showed a significant positive relationship between peroxidase activity and concentrations of Cu (*r* = 0.45 *p* = 0.089), As (*r* = 0.44, *p* = 0.095), and V (*r* = 0.54, *p* = 0.036) in 25F layers (Figure S2A).

### 3.5 Vertical Distribution of Moisture and Organic Matter

We observed no statistically significant differences in the organic matter content or moisture in vegetated site 25F (Figures S4A and S5A). Spearman correlation analysis showed that organic matter was significantly positively correlated with all nutrient cycling enzyme activities (cellobiohydrolase, *r* = 0.58, *p* = 0.025; l-leucine amino peptidase, *r* = 0.72, *p* = 0.003; phosphatase, *r* = 0.79, *p* = 0.0005) at site 25F (Figure S2). Conversely, no statistically significant differences were observed for the organic matter content or moisture with depth in barren site 25R (Figures S4B and S5B). Additionally, the organic matter content did not correlate with any enzyme activities in barren 25R.

## 4 Discussion

We determined the vertical heterogeneity of several soil chemical properties in the top 10 cm of soil, where the root density is the highest when plants are present. We found a distinct layer of heavy metals on the top of barren site 25R where no plants grow (Figure 2). We do not know why the metal cap exists, when the cap began to form, or how long it has been present. The metal distribution along the vegetated 25F soil layers was more uniform, and no metal accumulation was observed on the surface of 25F soil (no metal cap was present). This difference in metal distribution between the two sites could be related to the presence of roots in vegetated 25F soil where plant roots may have facilitated the mixing of the metals into the soil matrix.^44,45^ Conversely, in a barren soil, roots are not available to dilute and redistribute soil components.

Here, we have measured total soil metal concentrations. We acknowledge that metal toxicity is better understood when the bioavailability of metal contaminants is also known.^47^ Previous studies at the site have shown that As, Cr, Cu, and Zn are at least partially bioavailable and accumulate in plant stems and leaves.^45^ Ongoing studies are focused on the fraction of bioavailable metals in these soils, and the results of sequential extraction of metals and identification of metal complexes will be reported in due course. Another previous study done at LSP showed no significant variation in the concentrations of Zn, Cu, and Pb at the LSP site 25R from 1995 to 2015 in the upper 30 cm of the soil.^31^ There are multiple possible explanations for why the two sites 25F and 25R developed differently in terms of the vertical metal distribution over time, and we cannot distinguish among them based on the present day data that we have.

Heavy metal contamination can lead to soils becoming barren through changes in soil microbial communities. Gremion and co-workers examined bacterial communities in heavy metal-contaminated soils from a site in Ticino, Switzerland, which had been polluted with waste from septic tanks between 1960 and 1980 and contain high concentrations of Cd, Cu, and Zn.^46^ Analysis of 16S rDNA and 16S rRNA sequences showed that the rhizosphere and bulk soil of the metal-hyperaccumulating plant *Thlaspi caerulescens* housed unusual microbial communities. Actinobacteria were dominant in these contaminated soils, and the results showed that heavy metal pollution had led to a reduction in microbial diversity, a key factor in soil health and fertility. The decreased diversity may have been in part responsible for the observed decline in soil quality that contributed to the barrenness of the soil. We plan to do next-generation sequencing to assess whether the 0–2 cm layer on the surface of 25R soil (“the metal cap”) has lower microbial diversity as well.

Metals can also cause significant problems with germination and seedling growth by interfering with critical biological processes in plants. Baruah and co-workers showed that high concentrations of Cu, Pb, and Cd reduced seed germination rates significantly, with Cu being the most toxic.^48^ The toxicity has been reported to be due to reduction in amylase and protease activities, which are essential for breaking down food reserves in seeds.^49^ Additionally, metals can disrupt the cell membrane permeability and osmotic balance, further hindering germination.^50^ In seedlings, metals can lead to reduced chlorophyll biosynthesis, lower carbohydrate content, and impaired catalase activity, all of which contribute to poor seedling vigor and growth.^48^ Over time, this can result in soil becoming barren, as the accumulation of metals inhibits plant growth and seedling establishment.

During germination, seeds encounter the topmost soil layer, and the properties of this layer are expected to impact germination. Germination rates were significantly higher when the 25R soil was mixed than when its structure was intact (structured condition). These data indicate that the surface of 25R soil was not conducive to winter rye grass germination and that mixing the soil disturbed this surface layer and the vertical heterogeneity of 25R sufficiently to increase the germination rate. Soil properties, including contaminant concentrations, nutrient availability, and moisture levels, can vary with soil vertical depth. We are assuming that in the mixed soils, as a result of the homogenization, the differences in soil properties through depth were eliminated. When interpreting the data, we caution the reader to keep in mind that mixing the soil and returning it to the PVC pipe could have impacted soil compaction and possibly generated smaller aggregates. If so, then the altered particle size distribution could impact other soil properties, for example, microbial functioning.^51^ A previous study by Curtin and co-workers, however, examined the effects of physical disturbance on soil carbon and nitrogen mineralization and found that a physical disturbance associated with mixing the soil did not result in altered enzyme activities.^52^

We have previously shown that soils from site 25R have lower phosphatase, cellobiohydrolase, and l-leucine amino peptidase activities compared to 25F.^8,32,53^ These results agree with the results from this study, showing that 25R enzyme activities are lower than those in 25F across all depths (Figure 4). Site 25R is barren while 25F is vegetated, and the moisture holding capacity of 25R is lower than that of 25F (Section 2.1). As a barren soil, 25R receives fewer organic inputs both in decaying plant matter and in the form of root exudates and, not surprisingly, has a lower organic matter content.^32^ We noted a trend of decreasing enzyme activities with depth for soil cores from 25F. Accumulation of leaf litter and subsequent organic inputs at the surface of the soil may have contributed to the higher enzyme activities in the top 0–2 cm layers of vegetated 25F soil cores. Previous studies have also generally revealed a decrease in enzyme activities with depth.^54,55^ These studies, however, examined enzymatic function at larger depths of approximately a meter rather than resolving finer scale differences in the top 10 cm of the soil profile.^56^ We did not observe a similar trend for barren 25R soil where the enzyme activities did not vary systematically with depth. Despite the high metal concentration in the top 2 cm layer of 25R soil, the enzyme activities in this layer were not significantly depressed. This shows that, in poorly functioning soils, a further increase in metal concentrations does not necessarily lead to decreased soil nutrient cycling rates and that other soil properties can play a more significant role in limiting enzymatic activity.

Although both soils are heavy metal-contaminated, the presence of roots in 25F likely primes the soil such that the capacity of microorganisms to metabolically recycle nutrients or mitigate contaminant stress may be increased.^32,57^ The roots in 25F soil exude root exudates, which may provide nutrients to soil microbes, revitalizing them and possibly increasing their ability to synthesize and exude enzymes into the soil, even in the metal-contaminated brownfield environment. Additionally, some of the compounds in the root exudates may serve as siderophores to sequester metals in the 25F soil. The activities of the enzymes associated with nutrient cycling were very low in barren 25R layers.

In contrast, we detected substantial peroxidase activities in barren 25R soil (Figure 4H); for example, the peroxidase activity was 9.5 ± 4.2 μmol/(h g_dry soil_) in the 8–10 cm layer. Peroxidase activity in barren site 25R did not vary significantly with soil depth. Increased peroxidase production in plants caused by metal stress and subsequent peroxidase exudation to soils may contribute to these trends. Other studies have demonstrated a positive relationship between soil peroxidase activity and metal concentrations.^58^ Increased peroxidase activity may be a general plant response to the uptake of toxic metals; for example, peroxidase induction was found to increase with Zn and Cd concentrations in soils.^58−60^ Peroxidase enzymes produced by plants can be exuded into the soil, and increased peroxidase activity can be a protective response to oxidative stress, for example, cellular damage caused by active forms of oxygen including hydrogen peroxide.^61^

In most soils, decomposing plant residue lands on the surface of the soil, contributing to the higher organic matter content in the topsoil close to the surface. According to Rawls and co-workers, soils with a higher organic matter content tend to also have higher water retention.^62^ The significant positive correlations between organic matter and enzyme activities at site 25F suggest that the increased organic matter provides nutrients to soil microbes, and these microbes increasingly produce and exude enzymes into the soil. Conversely, the lack of significant differences in the organic matter content or moisture at the barren site 25R and the lack of correlations between organic matter and enzyme activities suggest that any debris that lands on the surface of the 25R soil is degraded slowly, or not at all, consistent with the low organic matter in the top layer. Hagmann and co-workers found that both sites 25R and 25F at Liberty State Park (LSP) were contaminated with significant amounts of anthropogenic materials, including volatile bituminous and anthracite coal, as well as tar and pitch-like substances.^28,30^ The authors also identified many fossil fuel biomarkers including polycyclic aromatic hydrocarbons, hopanes, steranes, and sesquiterpenes. While the overall organic contaminants did not significantly differ between the sites, site 25R had a lower yield of extractable organic matter compared to 25F. This suggests that the organic matter in 25R is less bioavailable or more tightly bound to the soil, which could result from and/or contribute to the barren conditions at 25R.

Determining the vertical distribution of soil components in postindustrial barren soils can support the management of urban brownfields. For example, discovering that a contaminated soil has a high metal concentration cap on its surface can motivate cost-effective options to facilitate germination, primary production, and increased soil enzymatic function. Cost-effective options may include removal of a thin topsoil layer or mixing of the topsoil with the layers below to prepare contaminated soils for plant growth. Introducing vegetation to industrial barrens is desirable because plant roots can distribute and dilute contaminants, emit exudates, and support an active rhizosphere microbial community, with all factors associated with improved soil function.^53,57,63^ Moreover, the presence of plants can stabilize contaminants and reduce their migration into groundwater.^64^ The increased germination rate measured in the mixed condition compared to the structured condition supports mixing the top 10 cm of similarly contaminated soils to dilute contaminants and redistribute soil nutrients.

## 5 Conclusions

We measured physical and chemical parameters along a 10 cm vertical soil gradient in contaminated vegetated and contaminated barren soils. These data showed statistically significant metal accumulation in the top 0–2 cm layer of the barren 25R site, forming a “metal cap”, while metal distribution was more uniform in the vegetated site 25F (Figure 2), likely due to the presence of roots. Winter rye grass germination was limited in the top layer of the barren soil 25R, where metals accumulated. Enzyme activities varied more significantly with depth in vegetated 25F, whereas in barren 25R soil, they remained consistently low. The soil moisture and organic matter content showed no significant variation with depth in either site. These findings illustrate the complex relationships among soil depth, contaminant concentrations, and microbial activity in brownfield soils and the important role that vegetation can play in improving soil function. Understanding vertical soil distribution in postindustrial soils can inform cost-effective strategies like removal of topsoil or soil mixing to improve soil function and revegetate urban brownfields.

## Supporting Information Available

Additional experimental results including metal concentration comparison, soil organic matter and moisture, Michaelis–Menten kinetics plot, and Spearman correlation matrix (PDF)
