# Supplementary material for: Brownfield Topsoil Vertical Heterogeneity: Implications for Germination and Soil Microbial Functioning
Source: ACS Omega. 2024 Sep 25;9(40):41544–54. doi: 10.1021/acsomega.4c05265 (PMC11465544; doi:10.1021/acsomega.4c05265)
Supplement: Supplementary file 1 — ao4c05265_si_001.pdf [file ao4c05265_si_001.pdf]

Supporting information

Soil metal concentrations (mg/kg)	Vegetated 25F (bulk)[a]	Vegetated 25F (core average)	Barren 25R (bulk)[a]	Barren 25R (core average)
Lead	7,145 ± 1,351	7,069 ± 1,288	20,302 ± 4,203	3,873 ± 3,108
Nickel	121.4 ± 13.8	116 ± 18	317.4 ± 62.5	76 ± 52
Cobalt	256.5 ± 46.5	237 ± 47	868.7 ± 178.8	178 ± 141
Copper	2,256 ±320	2,479 ± 374	7,165 ± 1512	2124 ± 1047
Zinc	14,435 ± 2845	9,224 ± 1849	41,271 ± 8526	6,896 ± 5566
Arsenic	630 ± 187	272 ± 50	1,162 ± 207	180 ± 134
Barium	533 ± 53	522 ± 74	1,467 ± 267	409 ± 214
Vanadium	70.7 ± 9.5	149 ± 24	77.3 ± 11.8	36 ± 10

Table S1 shows a comparison between individual metal concentrations in 25R and 25F soils. Both sites have the same metals present, the concentrations of some of the metals are higher in 25R while the concentrations of others are higher in 25F. [a]Bulk soil values were taken from Hagmann et al., 2019. The core averages shown were calculated as the averages of the concentrations of each metal in the five vertical layers (0-2, 2-4, 4-6, 6-8, and 8-10 cm). Standard errors are shown and reflect the variation among the layers; n=5.

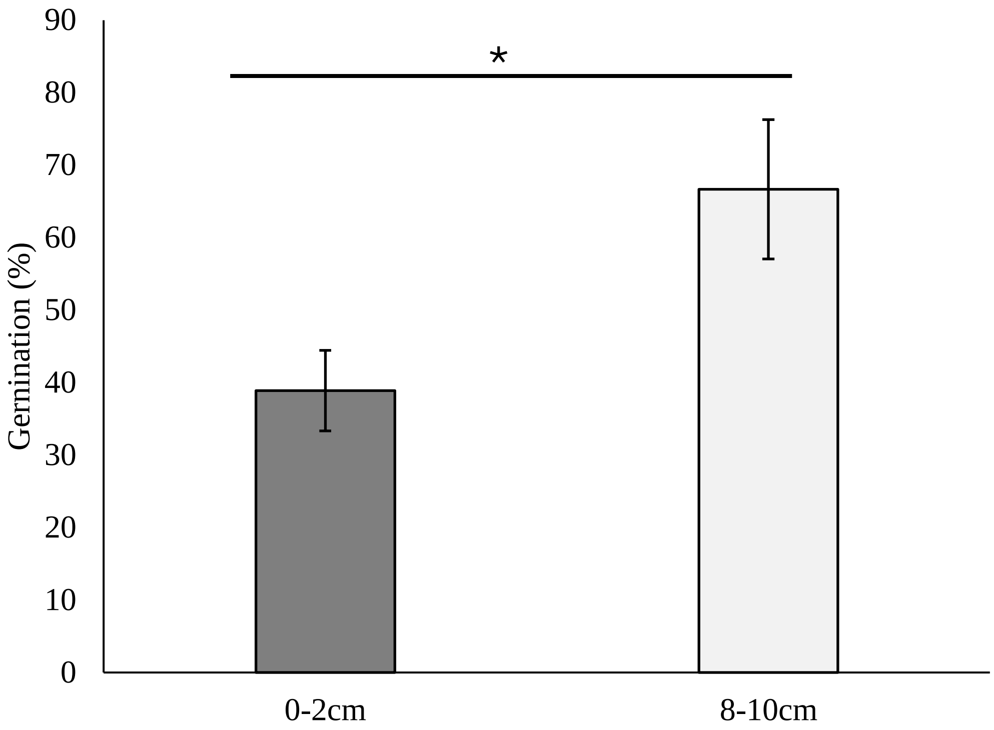

Figure S1: The germination rate for seeds planted in the top (0-2 cm) and bottom (8-10 cm) of 25R soil. The germination rate in the 0-2 cm layer (39 ± 6%) was lower than in the 8-10 cm layer (67 ± 10%) ($t = 2.5$, $df = 3.2$, p-value = 0.08). Standard error is shown, n=3.

Vegetated Site 25F

Barren Site 25R

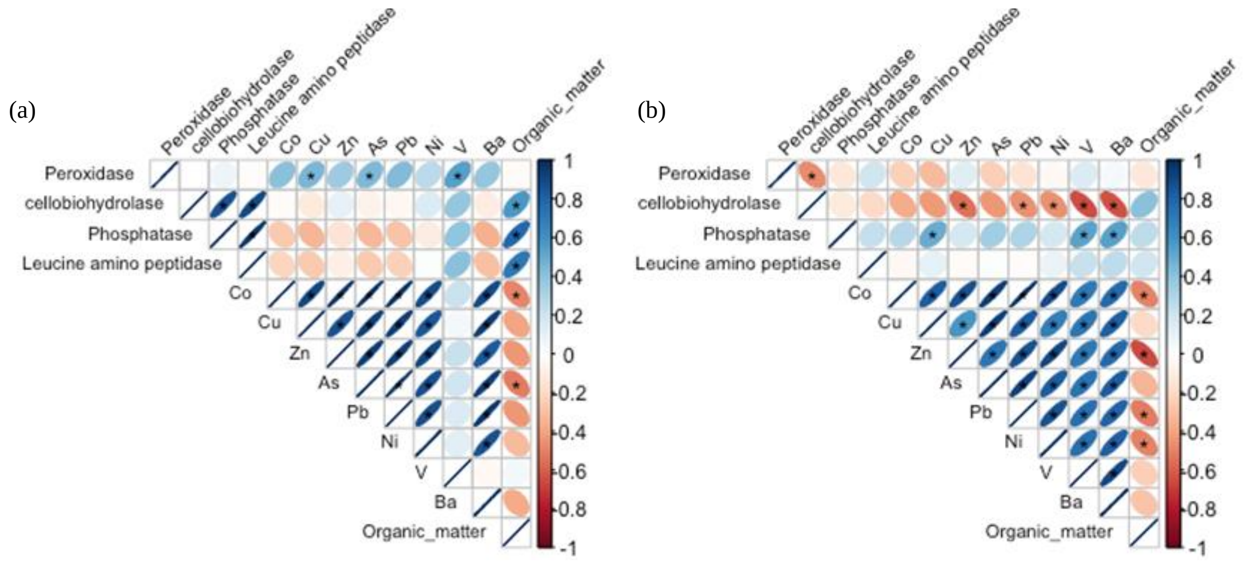

Figure S2. Spearman correlation matrix of relationships among enzyme activities, organic matter, and concentrations of inorganic elements for 25F and 25R. The figure was generated using the *Hmisc* package in R (version 3.6). Significant correlations are indicated with a star ($p < 0.1$).

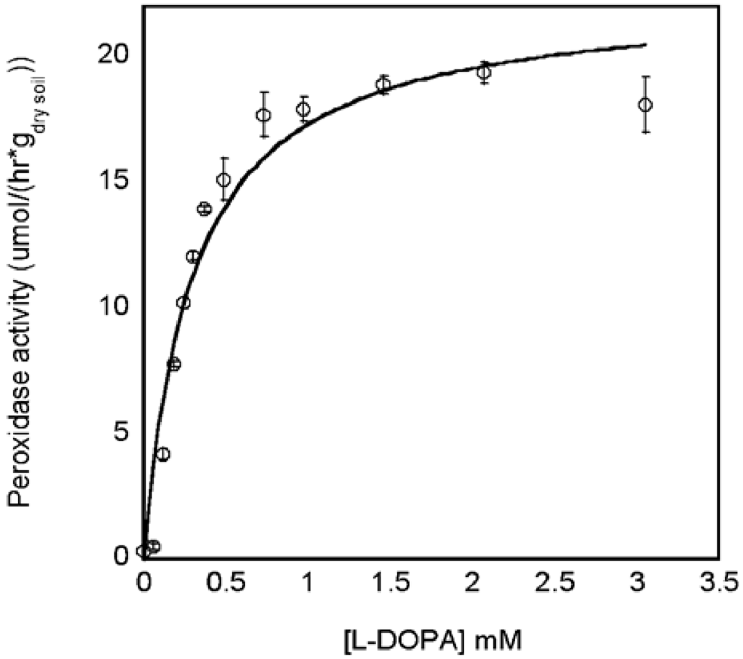

Figure S3: The relationship between peroxidase activity and substrate (L-DOPA) concentrations is shown for bulk vegetated 25F soil. Each data point represents an average of three measurements, and standard errors are shown. The data were fitted to the Michaelis-Menten equation, yielding a V_{max} of 22.5 ± 1.4 s^{-1} and a K_M of 0.29 ± 0.06 mM for L-DOPA.

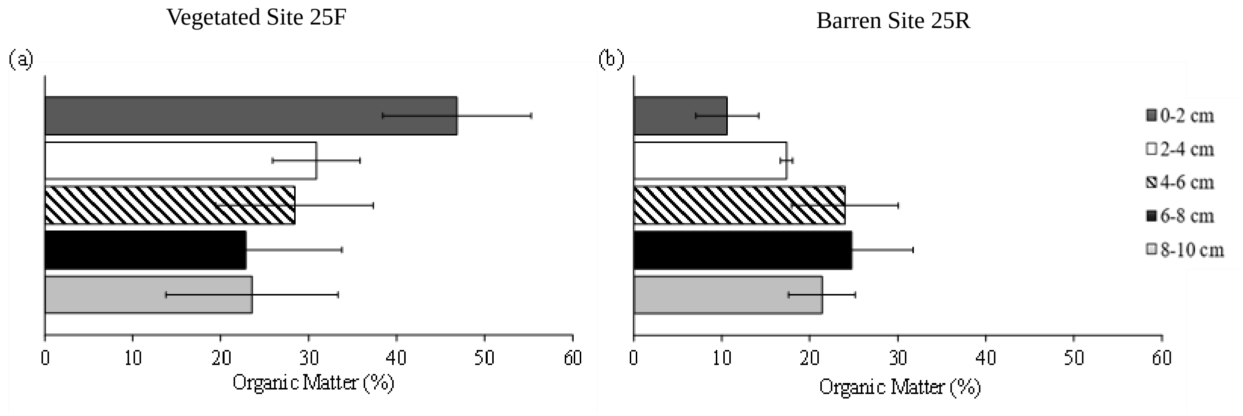

Figure S4. The figure illustrates the percentage of organic matter present in the soil in sites 25F (a) and 25R (b) for five different cross-sections. In (a) 25F, organic matter percentage is the highest in the 0-2 cm cross-section, decreasing deeper into the soil. In (b) 25R, organic matter percentage is the lowest in the top 0-2 cm cross-section and similar throughout the rest of core.

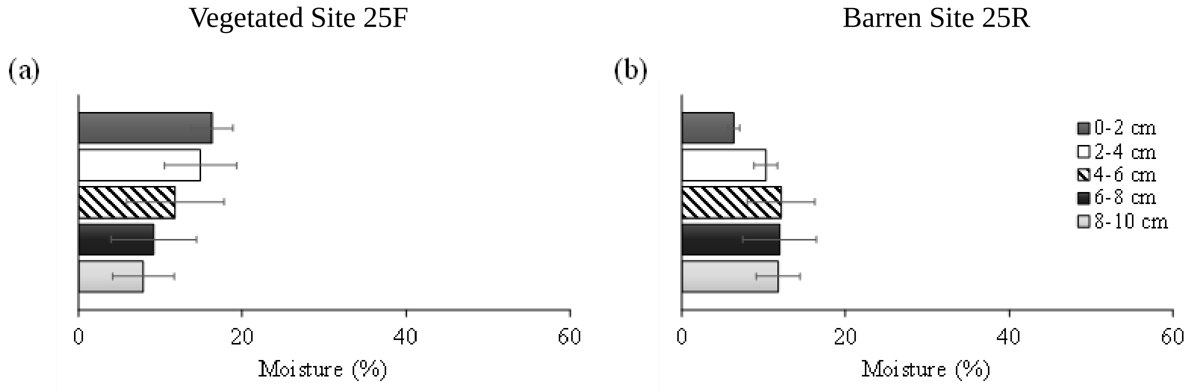

Figure S5. The figure illustrates the average percent moisture present in the soil in sites 25F (a) and 25R (b) for five different cross-sections. Error bars represent the standard error (n=3) for each measurement and no significant difference through depth was found for either soil. Moisture decreases with depth in 25F soil (a) and increases with depth in 25R soil (b).
